# The influence of different load distribution considering geometric error on the fatigue life of ball screw

**DOI:** 10.1038/s41598-024-60247-8

**Published:** 2024-04-26

**Authors:** Qin Wu, Jianxiong Li, Jun Liu, Xinglian Wang

**Affiliations:** 1 College of Mechanical and Electrical Engineering, Lanzhou University of Technology, Lanzhou, 730050 China; 2 CEPE, Centre for Mechanical Efficiency and Performance Engineering, University of Huddersfield, Huddersfield, HD1 3DH UK; 3 Mechanical and Electrical Operation and Maintenance Center, Lanzhou Petrochemical Company, Lanzhou, 730060 China

**Keywords:** Geometric error, Rotational moment, Load distribution, Fatigue life, Engineering, Mechanical engineering

## Abstract

The ball screw pair is a precision drive component that converts rotary motion into linear motion. In practical applications, because the feed system usually has rotational torque and geometric errors, it may increase the contact load of the ball screw pair and reduce the fatigue life. In order to study the influence of different loads and geometric errors on the maximum contact load and fatigue life of ball screw pairs, the following research work was carried out. Firstly, based on the composite load and rotational torque, the ball load distribution model is established, and the accuracy of the model is verified by finite element modeling analysis. Then, the influence of different composite loads, rotation times and geometric errors (including ball size error, lead error and raceway tooth profile error) on the ball load distribution is analyzed. Finally, the influence of compound load, rotating torque and geometric error on the fatigue life of ball screw pairs is studied. The results show that the rotational torque and lead error have a great influence on the load distribution and fatigue life of the ball, and the influence of lead error on load distribution is greater than that of dimension error and tooth profile error. The fatigue life of the ball screw pair with non-uniform load distribution is shorter than that of the ball screw pair with uniform load distribution.

## Introduction

The load distribution of the ball screw sub directly affects its load carrying capacity and stiffness. When the ball screw feed system is in operation, the size of the load bearing capacity of the balls in the raceway reflects the load bearing capacity of the entire ball screw sub to a certain extent. In the ball screw feed system, the loading condition of each ball depends on the structural parameters and material characteristics. However, in actual operation, the actual force on each ball in the ball screw subassembly is not uniformly distributed due to uncertainties in the machining and assembly process. For the ball screw sub with one end fixed and one end mounted. When the feeding system is working, the movement of the support end and the assembly error cause the screw to be subjected to extra rotating torque, which leads to uneven bearing of the balls in the raceway, and then affects the fatigue life of the ball screw sub. Therefore, it is important to study the actual load capacity of each ball in the raceway during the actual operation of the ball screw feed system.

Liu et al.^1^ analyzed the factors affecting the change of axial position of the raceway, and established and verified the calculated contact load model using C5Z10 ball screw vice as an example according to the relationship between the contact point of the ball and the raceway with the variation of contact deformation, and the experiment showed that the contact stress and contact angle both increased nonlinearly with the increase of axial load when a single load was applied, and the contact load of the ball in the nut raceway was significantly uneven, and the contact load near the bearing end was relatively greater. Hai et al.^2^ investigated the structure of the inner structure of the ball screw sub, investigated the internal load distribution of the ball screw sub based on Hertz contact theory, calculated the axial static stiffness based on the deformation of the inner contact of the ball screw sub, and verified the feasibility of the method by example analysis and finite element simulation. Luo et al.^3^ described the tilting components of contact deformation between the ball and raceway with quadratic numbers, indicated the raceway with parametric configuration, superimposed the effect of geometric tolerance, and conducted the solution for the contact load of the ball, and the investigation demonstrated that the contact load was influenced by the geometric tolerance, and with the enlargement of radial load, the contact load and contact deformation of the ball experienced nonlinear changes. Mei et al.^4^. a nonlinear dynamic model was established to study the periodic microwaving of a convex linear guide when subjected to a load in each of the five directions. For the precise description of the contact states of each ball, the calculation of the contact load for each ball was performed. Chen et al.^5^. investigated the influence of the geometric error of the ball screw assembly on the bearing performance of the balls in the raceway when a monaxial load is applied, and the study showed that when the geometric error of the ball screw assembly occurs, the positive error leads to the interference fit state between the balls and the raceway, the contact deformation of the balls is increased, and the phenomenon of uneven load on the balls is increased and the amplitude is greater. When the negative error results in the clearance fit between the ball and the raceway, the ball’s contact stress decreases, and stiffness decreases. Chen et al.^6^ investigate the relationship between the contact angle and the contact load between the ball and the raceway by numerical simulation, and the investigation shows that while the ball is in different locations in the raceway, the contact angle and the contact load between the ball and the raceway will increase with the change of the ball position, and the contact load is closer to the bearing on one side, and the differential stress between the balls increases with the increase of distance. Wang^7^ performed force and deformation analysis on the normal track section of the ball and track at the contact point, established a load distribution model of the ball screw joint, and analyzed the effect of linear prediction error on the contact load. The investigation showed that the load bearing ratio of the ball in the raceway was affected by the linear prediction error, and the ball in the middle raceway of the nut was loaded less and the ball at both extremities was loaded more.

Gong et al.^18^ proposed the concept and calculation method of fatigue elastic life based on the plastic contact deformation characteristics of ball screw under ultimate load condition. Zhao et al.^14^ studied the lubrication performance of ball screws under multi-directional loads, and proposed a thermal elastohydrodynamic lubrication contact model, which proved that the thermal effect would reduce the minimum oil film thickness, increase contact wear and shorten fatigue life. Zhao et al.^15^ studied the fatigue life of ball screw from the perspective of fatigue. The load distribution of double nut ball screw under axial load is analyzed. The influence of ball load distribution, curvature ratio and temperature rise on fatigue life is analyzed. Liu et al.^17^ studied the reliability of fatigue elastic life of ball screw. According to the existing fatigue elastic life model of screw, the reliability analysis model of fatigue elastic life is established based on uncertainty theory. The results show that among the factors affecting the reliability of fatigue elastic life of ball screw pair, the uncertainty of axial load has the least influence, followed by lead, ball diameter, pitch circle diameter and adaptation ratio, and the contact angle has the greatest influence. Zhen et al.^16^ used the external circulation ball screw pair as the research object, and established a calculation model that considers the ball size error, calculates the force of each ball and the life of the ball screw pair through mechanical analysis. The influence of size error on the force and fatigue life of balls under two conditions of single ball error and random error of all balls is studied. The maximum contact stress curve of balls is drawn, and the fatigue life of ball screw pair is calculated.

The above research mainly studies the load distribution and different influencing factors of fatigue life of ball screw pair under axial load, and only considers the influence of size error on ball force and fatigue life. The established ball contact load model generally assumes that all balls in the ball cycle sequence are subjected to similar loads, ignoring the variability of each ball load. Considering the variability of ball bearings, most of the analysis only analyzes the load of the ball screw pair when a single or composite load is applied, without in-depth investigation of the influence of different geometric errors on the load distribution and fatigue life of the ball. In summary, researchers mainly focus on the influence of single load and error on the fatigue life of ball screw pairs, while there are relatively few studies on considering different load distribution and geometric errors. Therefore, it is of great significance to accurately calculate the actual load and fatigue life of ball screw pairs in the raceway to improve the performance of ball screw pairs.

## Ball screw load distribution model of ball screw

### Analysis of ball screw force

In this paper, the mechanical modeling of ball screw subsets commences with the introduction of the following assumptions:

1. Investigate contact deformation in the range of elastic deformation;
2. Neglecting the effect of centrifugal forces.

**Figure 1:**
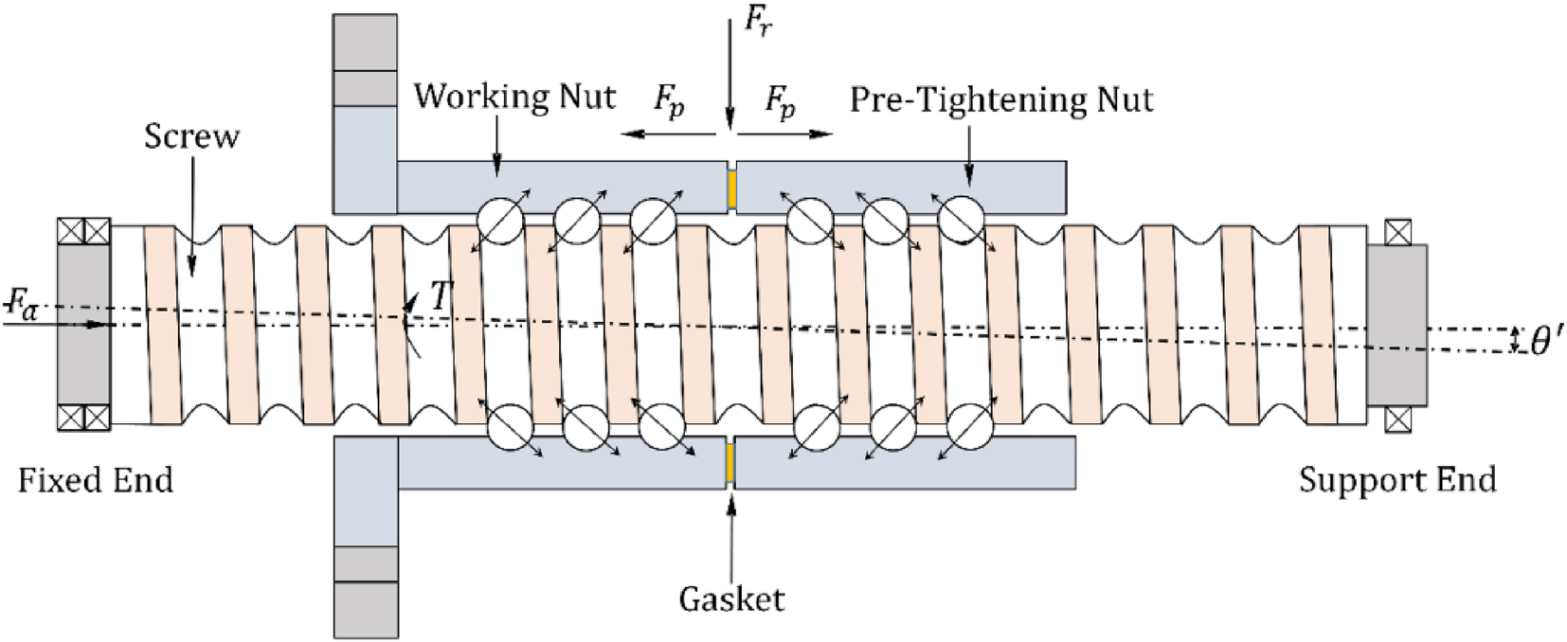
Schematic diagram of double nut preload ball screw structure.

The corresponding position of the screw and nut with external load and preload is shown in Fig. 1. When the installation method is fixed at one end and supported at the other, and due to assembly error, the actual screw axis will be inclined downward or upward relative to the theoretical screw axis, thus forming an angle, resulting in the subassembly of the ball screw being subjected to additional rotational torque^8^ T is the additional rotational torque on the screw, and $\theta^{\prime}$ is the deflection angle.

During the operation of the ball screw, the nut is fixed to the base and the screw rotates, subject to axial load F_a_, radial load F_r_ and preload F_p_. According to the force analysis of the nut, the left end nut is the working nut and the right end nut is the preload nut. Taking the working nut as the object of investigation, according to the axial load balance and literature^19^, the load relationship between the load of all balls and the external axial load can be expressed as:

1 $$F_{a} = \mathop \sum \limits_{i = 1}^{z} p_{i} sin\beta_{i} cos\lambda$$

**Figure 2:**
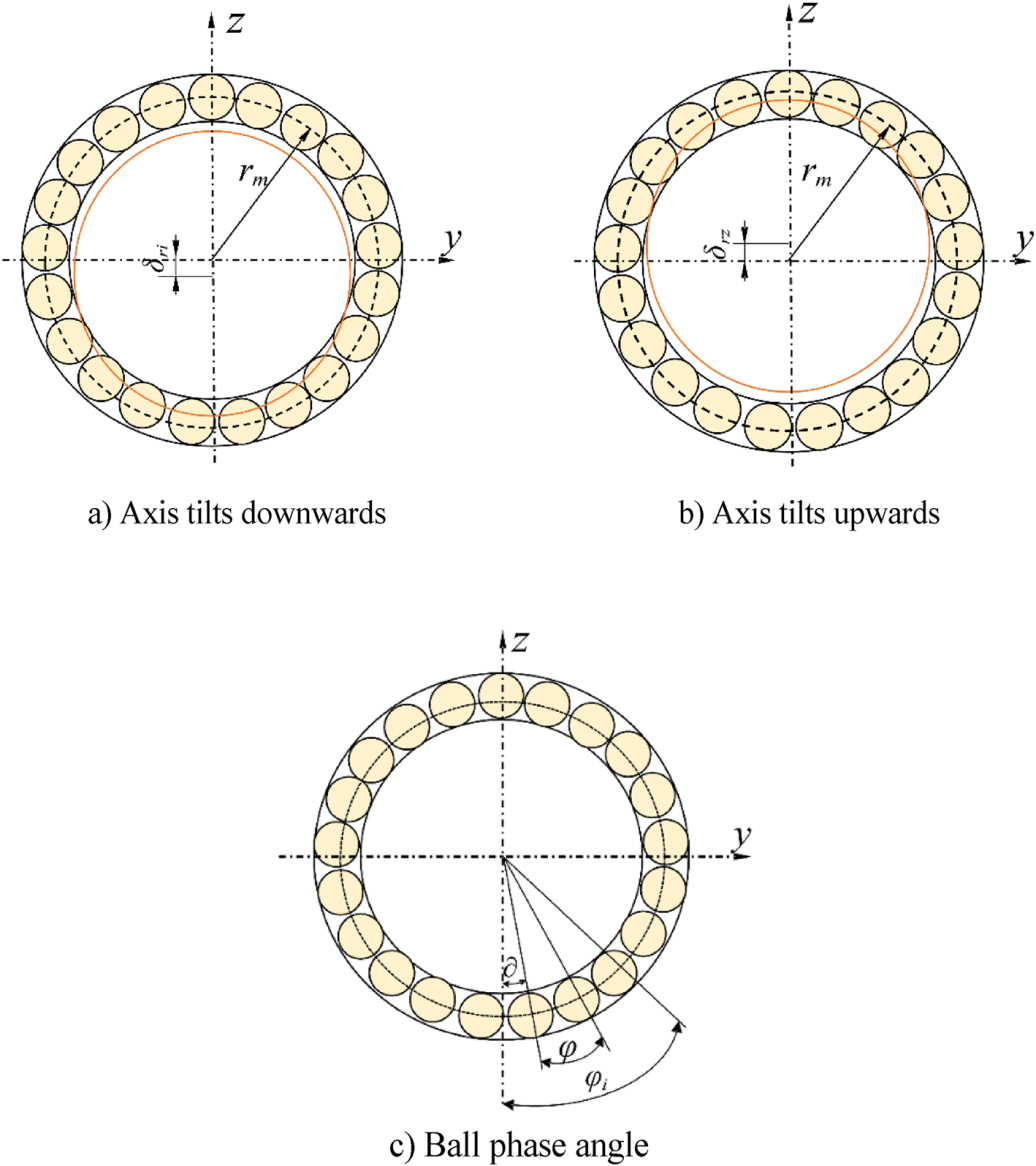
Axial view of the track.

In the formula:$p_{i}$ is the normal force of each ball; z is the actual effective bearing ball number; $\beta_{i}$ is the contact angle of the first i-ball; $\lambda$ is the lead angle;

The schematic diagram of the phase angle of the balls in the ball screw sub is shown in Fig. 2c, and the position of any individual ball in the raceway can be described by the position angle relationship Eq. (2). *v* is the position angle of the first ball in the raceway; *ψ i*s the angle between two adjacent balls.

2 $$\varphi_{i} = \left( {i - 1} \right)\varphi + \partial$$

Due to the downward or upward inclination of the actual screw axis relative to the theoretical axis, a certain angle is formed. Figure 2a shows the upward deflection of the screw shaft, and Fig. 2b shows the downward deflection of the screw shaft. Due to the upward or downward deviation of the axis, when the ball screw pair is operated, it is affected by additional rotational torque. That is, the influence of the deflection angle is replaced by the action of torque.

According to the position angle relationship between the ball in the raceway and the axial perspective analysis, the radial displacement of any one ball in the raceway can be expressed as follows:

3 $$\delta_{ri} = \delta_{r} {\text{cos}}\varphi_{i}$$

According to the radial load balance, the relationship between the load on the ball and the external radial load can be expressed as follows:

4 $$F_{r} = \mathop \sum \limits_{i = 1}^{z} p_{i} {\text{cos}}\beta_{i} {\text{cos}}\varphi_{i}$$

The instantaneous equilibrium equation is as follows:

5 $$T = \mathop \sum \limits_{i = 1}^{z} p_{i} {\text{sin}}\beta_{i} {\text{cos}}\lambda \left( {r_{m} - r_{b} {\text{cos}}\varphi_{i} } \right)$$

### Ball contact deflection analysis

**Figure 3:**
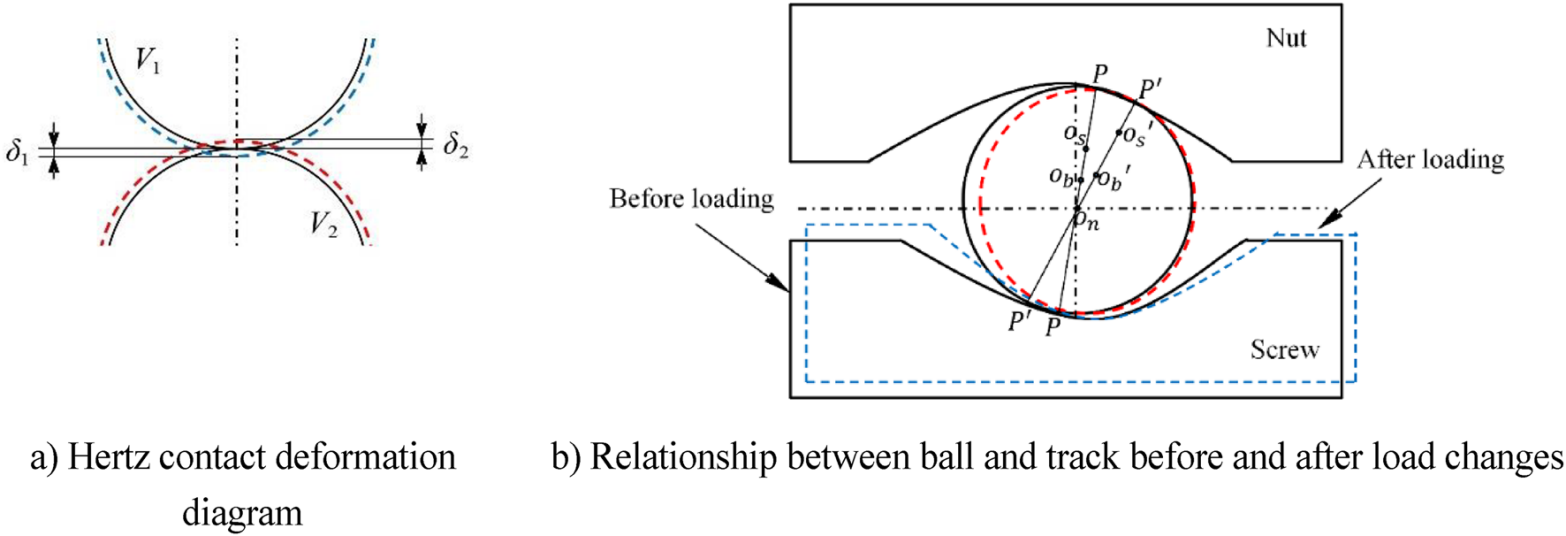
Diagram the change of ball and track before and after loading.

The Hertz contact deformation of two elastomers in the elastic deformation phase from unloaded (solid part) to loaded (dashed part) is shown in Fig. 3a. The deformations are $\delta_{1}$ and $\delta_{2}$ respectively . Then the contact deformation in the elastic deformation phase can be expressed as ${{ \updelta }}$, ${\updelta } = \delta_{1} + \delta_{2}$.

For the analysis of the ith ball, the distance between the two raceway curvature centers $O_{S}$ and $O_{n}$ when not under load as shown by the solid line in Fig. 3b is $A_{O}$:

6 $$A_{0} = O_{s} O_{n} = R_{n} + R_{s} - D$$

When the ball screw vice is not subjected to external load only in the preload, the normal contact load between the ball and the raceway is:

7 $$p_{ni} = p_{si} = \frac{{F_{p} }}{{z{\text{sin}}\beta_{0} {\text{cos}}\lambda }}$$

where, $P_{ni}$ is the contact load of ball and nut race in the direction of contact normal; $P_{si}$ the contact load of ball and screw joint race in the direction of contact normal; $\beta_{o}$ the contact angle under preload.

where $O_{b}^{\prime }$ is the center position of the ball after contact deformation due to the change of geometric position; $O_{s}^{\prime }$ is the center position of the curvature of the screw race after axial contact deformation due to the change of geometric position.

The rotation angle $\theta$ of the screw in the normal plane and the rotation angle $\theta^{\prime}$ in the axial plane are transformed into each other as follows^9^:

8 $$tan\theta = tan\theta^{\prime}cos\lambda$$

Under the action of the coupling load and the additional rotating moment, the ball screw sub-deformation occurs in contact as shown in the dashed line in Fig. 3b, and the center of curvature of the screw raceway is shifted, $O_{s}$ in axial displacement under axial load, $\delta_{X} = \delta_{a} /\cos \lambda$ in radial displacement under radial load, and $\delta_{ri} = \delta_{r} \cos \varphi_{i}$ in rotational displacement under the action of the moment $R_{i} = \theta R_{i}^{*}$ (where $R_{i}^{*} = r_{m} + \left( {r_{s} - r_{b} } \right)\cos \beta_{o}$). The deformation under load due to the change in geometric position can be expressed as:

9 $$A_{0}^{\prime } = \sqrt {\left( {A_{0} {\text{cos}}\beta_{0} + \delta_{ri} } \right)^{2} + \left( {A_{0} {\text{sin}}\beta_{0} + \left( {x_{m} + \delta_{m} } \right){\text{cos}}\lambda + \frac{{\delta_{a} }}{{{\text{cos}}\lambda }} + \theta R_{i}^{*} } \right)^{2} }$$

where, $\left( {x_{m} + \delta_{m} } \right)\cos \lambda$ indicates the axial displacement in the normal plane between the displacement produced by the preload shim and the preload deformation due to the thickness of the shim itself squeezing each other with the nut in space when the preload shim is used for preload.

The contact deformation of the ball may be expressed by the contact deformation relationship between the ball and the raceway^10^, and the contact deformation of any ball is $\delta_{i}$.

10 $$\delta_{i} = A_{0}^{\prime } - A_{0}$$

The contact angle between the ball and the raceway at the contact point is affected by the contact deformation, resulting in a change in the geometric parameters of the contact area. The contact angle between the ball and the raceway after the contact deformation occurs is:

11 $$\beta_{i} = {\text{sin}}^{ - 1} \left( {\frac{{A_{0} {\text{sin}}\beta_{0} + \left( {x_{m} + \delta_{m} } \right){\text{cos}}\lambda + \theta R_{i}^{*} + \frac{{\delta_{a} }}{{{\text{cos}}\lambda }}}}{{A_{0}{\prime} }}} \right)$$

### Modeling load distribution when geometric errors are not considered

From the analysis of Hertz contact theory, it follows:

12 $$p_{i} = K\delta_{i}^{\frac{3}{2}}$$

13 $$\frac{1}{{E^{\prime}}} = \frac{{1 - \mu_{1}^{2} }}{{E_{1} }} + \frac{{1 - \mu_{2}^{2} }}{{E_{2} }}$$

14 $$K = \left[ {\frac{{2k_{s} \left( e \right)}}{{\pi m_{as} }}\sqrt[3]{{\frac{1}{32}\left( {\frac{3}{{E^{{{\prime} }} }}} \right)^{2} {\Sigma }\rho_{s} + \frac{{2k_{n} \left( e \right)}}{{\pi m_{an} }}\sqrt[3]{{\frac{1}{32}\left( {\frac{3}{{E^{{{\prime} }} }}} \right)^{2} {\Sigma }\rho_{n} }}}}} \right]^{{\frac{ - 3}{2}}}$$

Combining cubic equations, the equilibrium equation of the working nut can be obtained as follows:

15 $$F_{r} = \mathop \sum \limits_{{i = 1}}^{z} k\delta _{i}^{{\frac{3}{2}}} \left( {\frac{{A_{0} {\text{cos}}\beta _{0} + \delta _{{ri}} }}{{A_{0}^{\prime } }}} \right){\text{cos}}\varphi _{i}$$

16 $$F_{r} = \mathop \sum \limits_{{i = 1}}^{z} k\delta _{i}^{{\frac{3}{2}}} \left( {\frac{{A_{0} {\text{cos}}\beta _{0} + \delta _{{ri}} }}{{A_{0}^{\prime } }}} \right){\text{cos}}\varphi _{i}$$

17 $$T = \mathop \sum \limits_{i = 1}^{z} k\delta_{i}^{\frac{3}{2}} \left( {\frac{{A_{0} {\text{sin}}\beta_{0} + \left( {x_{m} + \delta_{m} } \right){\text{cos}}\lambda + \theta R_{i}^{*} + \frac{{\delta_{a} }}{{{\text{cos}}\lambda }}}}{{A_{0}^{\prime } }}} \right){\text{cos}}\lambda \left( {r_{m} - r_{b} } \right){\text{cos}}\varphi_{i}$$

### Modeling load distribution when geometric errors are considered

**Figure 4:**
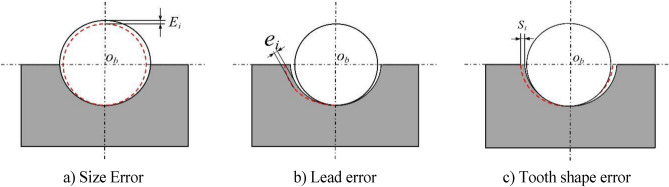
Geometric error and the relationship between ball position and raceway.

As shown in Fig. 4a is the dimensional error of the ball, Fig. 4b is the lead error of the screw, and Fig. 4c is the tooth shape error of the raceway^11^. Since the law of action of the ball size error and the raceway tooth shape error are similar, the raceway tooth shape error is equal to the ball size error. Then, since the presence of the screw guide error increases or decreases the deformation of the screw in the axial direction with respect to the nut, it can be considered the amount of deformation generated by the screw along the axial direction. The presence of the error causes the structural parameters of the ball screw pair to change, causing the load on the balls to change.

The effect of geometric errors is studied in depth and the different geometric errors are subdivided. Considering the effect of subdivided geometric errors on ball contact deformation, the contact deformation is corrected as follows:

18 $$\delta_{i} = \sqrt {(A{\text{cos}}\beta_{0} + \delta_{ri} )^{2} + \left( {A{\text{sin}}\beta_{0} + \left( {x_{m} + \delta_{m} + S_{i} } \right){\text{cos}}\lambda + \frac{{\delta_{a} }}{{{\text{cos}}\lambda }}} \right)^{2} } - A_{0} + E_{Ri}$$

Order:

19 $$A_{L} = \sqrt {(A_{0} {\text{cos}}\beta_{0} + \delta_{ri} )^{2} + \left( {A_{0} {\text{sin}}\beta_{0} + \left( {x_{m} + \delta_{m} + S_{i} } \right){\text{cos}}\lambda + \frac{{\delta_{a} }}{{{\text{cos}}\lambda }} + \theta R_{i}^{*} } \right)^{2} }$$

When subjected to load, the structural parameters on the local contact surface change, while the contact angle between the ball and the raceway affected by the error can be corrected as follows:

20 $$\beta_{i} = {\text{sin}}^{ - 1} \left( {\frac{{A_{0} {\text{sin}}\beta_{0} + \left( {x_{m} + \delta_{m} + S_{i} } \right){\text{cos}}\lambda + \theta R_{i}^{*} + \frac{{\delta_{a} }}{\cos \lambda }}}{{A_{L} + E_{Ri} }}} \right)$$

The correction of the contact deformation and the contact angle under the influence of geometric errors can be obtained from Eqs. (12), (18) and (20) for correcting equilibrium equations of forces and moments under the influence of errors.

21 $$\left\{ {\begin{array}{l} {F_{a} = \mathop \sum \limits_{{i = 1}}^{z} k\delta _{i}^{{\frac{3}{2}}} \left( {\frac{{A_{0} {\text{sin}}\beta _{0} + \left( {x_{m} + \delta _{m} + S_{i} } \right){\text{cos}}\lambda + \theta R_{i}^{*} + \frac{{\delta _{a} }}{{{\text{cos}}\lambda }}}}{{A_{L} + E_{{Ri}} }}} \right)cos\lambda } \\ {F_{r} = \mathop \sum \limits_{{i = 1}}^{z} k\delta _{i}^{{\frac{3}{2}}} \left( {\frac{{A_{0} {\text{cos}}\beta _{0} + \delta _{{ri}} }}{{A_{L} + E_{{Ri}} }}} \right)cos\varphi _{i} } \\ \end{array} } \right.$$

## Numerical simulation and analysis

Based on the analysis of elastic contact theory, a three-dimensional model is established to analyze the contact stress between the ball and screw race and nut race through finite element simulation.

### Simulation modeling

Take the ball screw 3210-4, for example. The ball flow path circuit includes an inverter that connects the start and end of the ball flow path, with a total of 76 balls. There are 64 balls under load and 12 balls in the inverter are not under load. The structural parameters of the ball screw sub are shown in Table 1.

**Table 1:** Ball screw performance parameters.

$D\left( {{\text{mm}}} \right)$	$d_{b} \left( {{\text{mm}}} \right)$	$L_{p} \left( {{\text{mm}}} \right)$	$r_{s} \left( {{\text{mm}}} \right)$	$D_{o} \left( {{\text{mm}}} \right)$	$\lambda (^{0} )$
6.34	4.763	8	2.477	32	4.55

$\beta_{0} \left( o \right)$	$\mu_{1} /\mu_{2}$	E(Pa)	Z	N	$n_{j} \left( {rmin^{ - 1} } \right)$
45	0.3	$2.1 \times 10^{11}$	76	2.5	600

According to the structural parameters in Table 1, the three-dimensional model of the ball screw sub is established by using drawing software for the analysis of the contact load between the ball and the raceway.

In order to improve the calculation efficiency, the 3D model of the ball screw sub is somewhat simplified^12^. The mesh size of the screw and nut is about 1 mm, and the fine mesh size of the ball is about 0.2 mm.

### Creating contact pairs with constraints imposed

The material parameters of each component in the ball screw sub are shown in Table 2.

**Table 2:** Ball screw material parameters.

Structure	Ball	Screw	Nut
Material	Gr15SiMn	GGr15	GGr15

### Simulation results analysis

**Figure 5:**
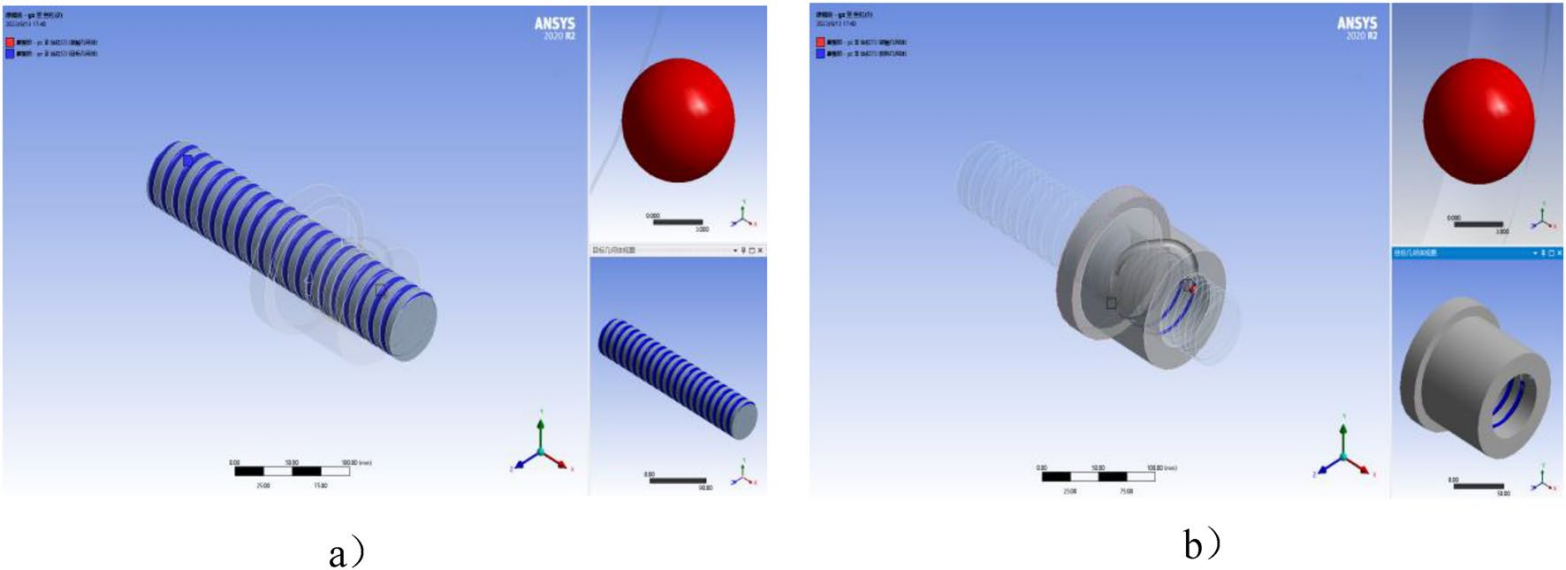
Ball and raceway contact surface setting.

The contact surface between the ball and the raceway is shown in Fig. 5. In order to make the analysis of the ball screw sub close to the actual situation, the contact form between the ball and the raceway is set to friction contact, and the friction coefficient is set to 0.05 for stable solution, and all other settings are controlled by the program. In addition, friction contact and penetration parameters require accurate surface characteristics and environmental condition data, which may not be easy to obtain. And adding more information may not significantly improve the accuracy or reliability of the model, so the simplified model is selected.

Apply a rotating sub to the screw relative to the ground and a moving sub to the nut relative to the screw. The load is applied in two steps, the first step applies gravitational acceleration to the entire ball screw sub, the second step applies preload, axial load, radial load, and torque to the screw to the nut.

**Figure 6:**
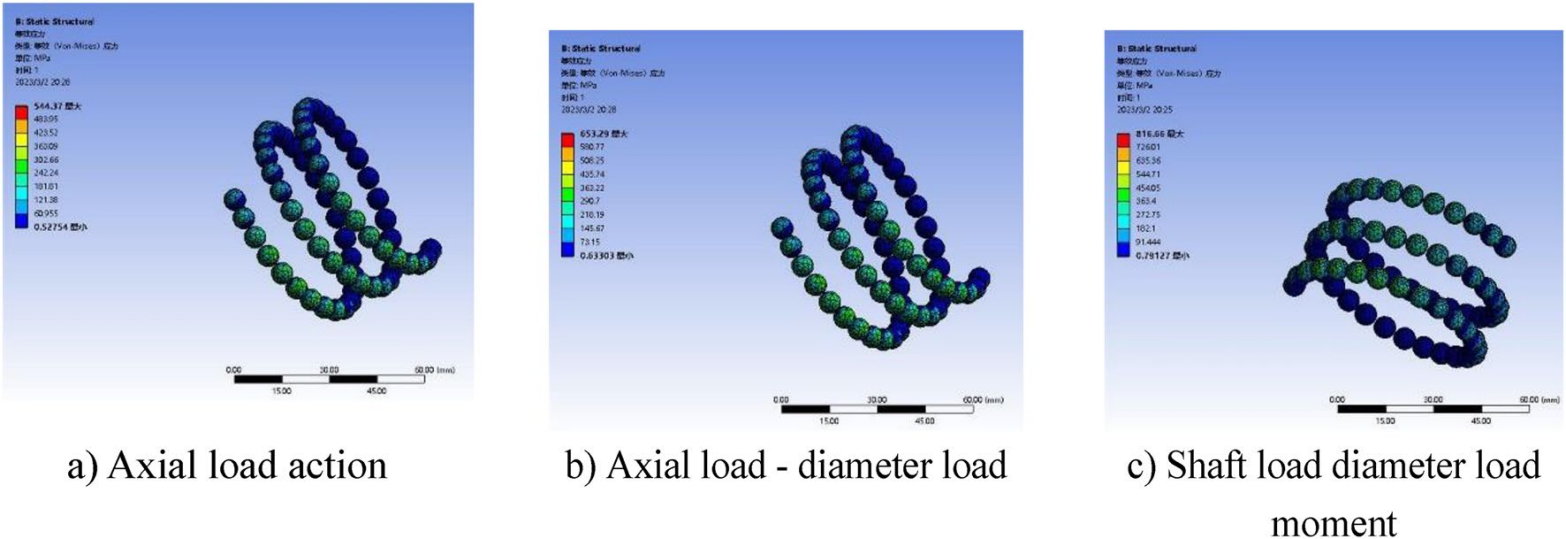
Equivalent ball contact stress cloud with F = 2500 N and Fp = 1000 N.

**Figure 7:**
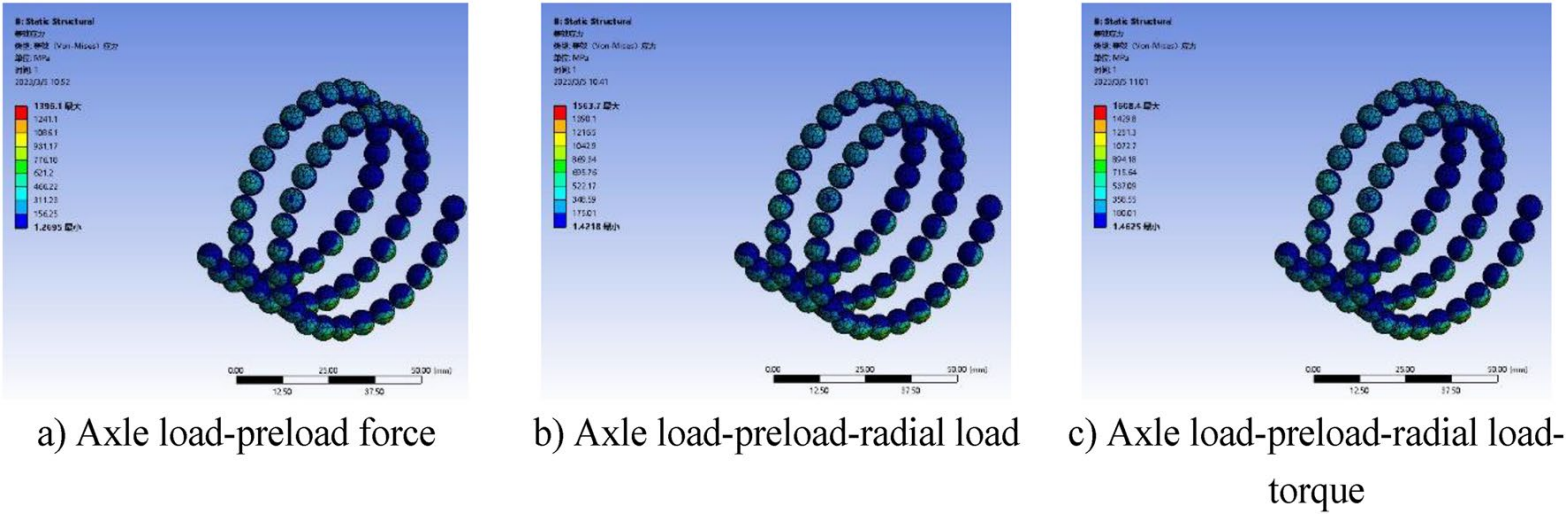
F = 4000 N, Fp = 1000 N ball equivalent contact stress cloud.

Through finite element simulation, the equivalent ball contact stress under different loading methods is obtained as shown in Figs. 6 and 7.

Figure 6a shows that when subjected to axial load and preload. According to the equivalent stress cloud diagram of the ball, the maximum equivalent stress is ${\upsigma }_{{{\text{max}}}} = 544.37\;\;{\text{MPa}}$. Figure 6b is the equivalent stress cloud diagram of the ball when the radial load is applied on the basis of Fig. 6a. It can be seen from Fig. 6b that the maximum equivalent stress is ${\upsigma }_{{{\text{max}}}} = 653.27\;\;{\text{MPa}}$. Figure 6c is the equivalent stress cloud diagram of the ball when it is subjected to additional rotational torque on the basis of Fig. 6b. It can be seen from Fig. 6b that the maximum equivalent stress is ${\upsigma }_{{{\text{max}}}} = 816.66\;\;{\text{MPa}}$.

Figure 7a shows the equivalent stress cloud of the ball under axial load and preload. The maximum equivalent stress is ${\upsigma }_{{{\text{max}}}} = 1396.1\;\;\;{\text{MPa}}$. Figure 7b is the equivalent stress cloud diagram of the ball when the radial load is applied on the basis of Fig. 7a. The maximum equivalent stress is $1563.7\;\;{\text{MPa}}$. Figure 7c is the equivalent stress cloud diagram of the ball when it is subjected to additional rotational torque on the basis of Fig. 7b. The maximum equivalent stress is ${\upsigma }_{{{\text{max}}}} = 1608.4\;\;{\text{MPa}}$.

According to Figs. 6 and 7, the equivalent force analysis of the ball can be seen. With the single load action, the coupling load action to the instantaneous action, the equivalent contact stress of the ball subjected to change, the equivalent contact stress of the ball gradually increased.

**Figure 8:**
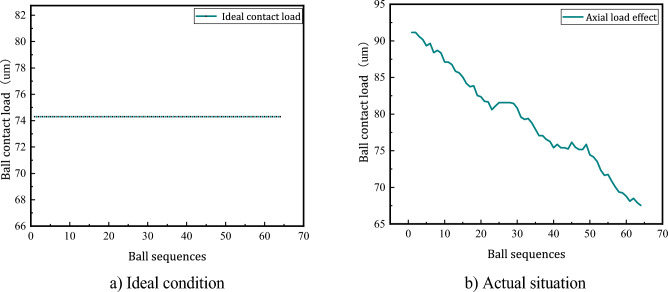
Ball load distribution law when axial load is applied.

Figure 8a is the distribution of ball contact load under ideal conditions. The load of each ball in the raceway is the same, and is not affected by the ball's position in the raceway. Figure 8b shows the distribution of ball loads when considering actual working conditions. From the analysis of Fig. 8b, it can be seen that the first ball is subjected to the maximum normal load. Due to the different positions of the ball in the raceway, the contact load of the ball gradually decreases with the increase of the distance between the ball and the preload shim according to the different distances between the ball and the preload shim.

**Figure 9:**
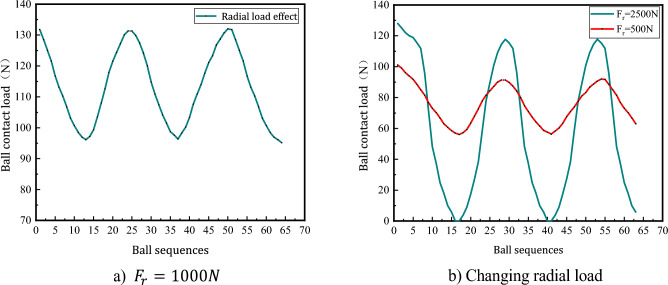
Load distribution law of the ball when radial load is applied.

Figure 9a shows the trend in ball load variation when radial load is applied based on axial load action. That is, when the radial load is applied on the basis of Fig. 8b, the load bearing change of the ball is parabolic and shows periodic change when the radial load is applied, and the maximum contact load of the ball also changes periodically. Figure 9b is the change trend of the load on the ball when the radial load is applied. When the applied radial load changes in a small range, the ball load also changes in a small range. The load gap between the balls is relatively small and changes uniformly in a certain range; when the applied radial load is larger, the load change amplitude of the ball is larger, the difference in contact load between the balls is larger; when the applied radial load is too large, it will cause the separation between part of the ball and the raceway, and is not subjected to the load.

**Figure 10:**
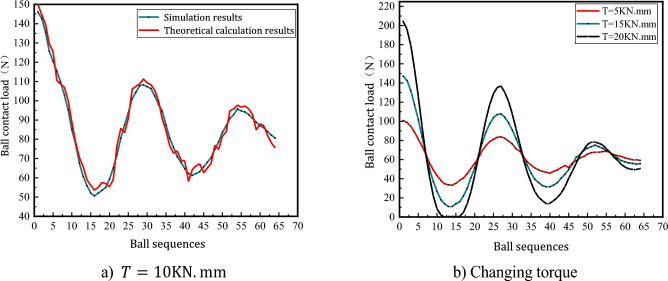
Ball load distribution law when rotating torque is applied.

Figure 10a shows the load distribution of the ball when subjected to additional torque action based on the coupling load action, i.e. the torque action is applied based on Fig. 9a. When subjected to the action of additional rotation time, the displacement of the ball in the radial direction is changed, the load bearing of the ball in the raceway changes periodically with increasing amplitude with the position angle, the load amplitude of the ball is gradually reduced to a certain range, and the load gap between adjacent balls is large.

From the analysis of Fig. 10a, it can be seen that the theoretical calculation results and the ball contact load obtained from the simulation experiment have the same trend of change, and the simulation results are greater than the theoretical calculation contact load on some balls, and the maximum error does not exceed 10%, which verifies the rationality of the ball load distribution model without considering the influence of error.

Figure 10b shows that when the coupling load is constant to change the rotational force, the contact load of the balls is different due to the different positions of the balls in the raceway. The action of the rotating moment makes part of the ball and the raceway separated uncontacted, and part of the ball's contact load is zero.

Through the above analysis, the load distribution law of the ball screw sub is compared when different external loads are applied. As the unevenness of the load on the balls gradually increases when acting from single load, coupling load to rotational moment, the contact load on the same ball also changes.

## Fatigue life analyses

### Effect of ball load distribution on ball screw fatigue life

When the ball screw sub -operates, the ball moves from entering the raceway contact area to leaving the raceway contact area, subject to periodic contact stress. According to the strength conditions of the contact theory, the contact stress formula can be obtained:

22 $$\sigma_{max,i} = \alpha_{\sigma } \sqrt[3]{{p_{max,i} E_{2}^{2} \left( {\frac{{r_{n} - r_{b} }}{{r_{n} r_{b} }}} \right)^{2} }}$$

where,$P_{max,i}$ is the maximum normal contact load on the $i$th ball; $\alpha_{\sigma }$ is the stress factor of the external recirculating ball screw.

The maximum contact stress between the ball and the raceway is obtained from the ball load distribution model, and the fatigue life of the ball is calculated. The exponential equation of the material fatigue curve^13^:

23 $$N_{H} = \frac{{\sigma_{0}^{m} N_{0} }}{{\sigma^{m} }}$$

where: $N_{H}$ is the number of fatigue life cycles; ${\sigma }$ is the corresponding contact stress; $\sigma_{o }$ is the cycle base; $N_{O}$ is the corresponding contact fatigue limit. When the hardness is HRC = 600,$\sigma_{O} = 2450\;\;\left( {{\text{N/mm}}^{{2}} } \right)$, $N_{o} = 10^{8}$, m = 8.

**Figure 11:**
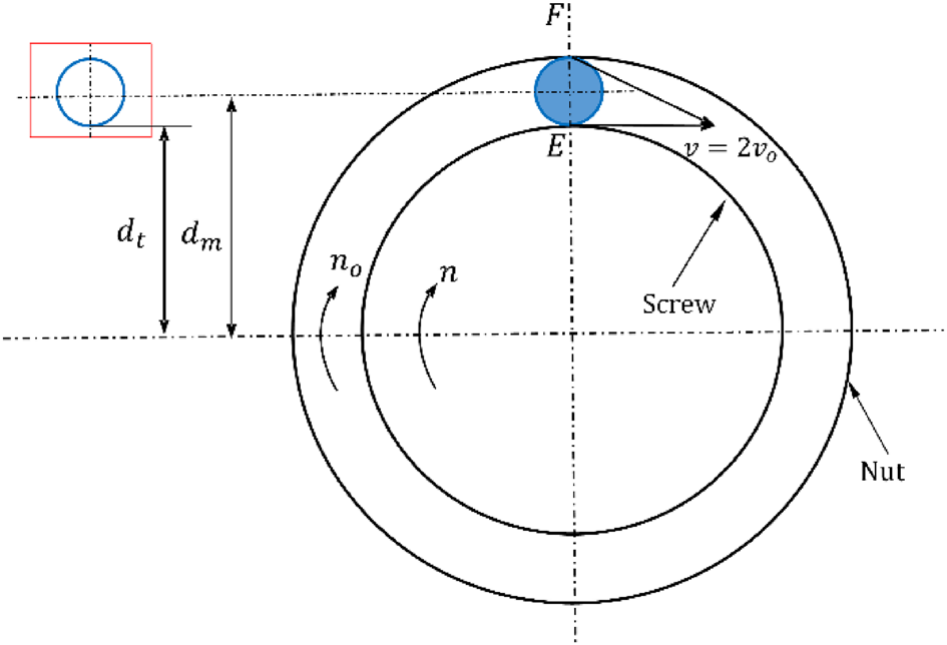
Single ball movement diagram.

The analysis of the motion of a single ball is shown in Fig. 11. When the screw rotates at speed $N_{j}$, the linear velocity at the contact point F is $V_{j}$, and the linear velocity at the center of the ball is $V_{D}$. The rotational speed of the ball can be expressed as:

24 $$V_{g} = \frac{{N_{j} }}{{2D_{0} }}\left( {D_{0} - D{\text{cos}}\beta_{0} } \right)$$

The ball around the screw can also rotate at the same time, and the rotation speed is set to $v_{h}$, at the contact point E ball and raceway phenomenon no relative sliding, then the ball and raceway at the contact point of the relative linear velocity equal, the ball rotation speed can be expressed as follows:

25 $$V_{h} = \frac{{N_{j} }}{{2D_{0} D}}\left( {D_{0}^{2} - D^{2} {\text{cos}}\beta_{0}^{2} } \right)$$

When the ball rotates every revolution, a certain point on the ball contacts the track twice, the number of cycles corresponding to the fatigue life is calculated by the exponential equation of the material fatigue curve, according to^20^ and finally the fatigue life *M* is expressed in hours:

26 $$M = \frac{{\sigma_{0}^{m} N_{0} D_{0} D}}{{60\sigma^{m} N_{j} \left( {D_{0}^{2} - D^{2} {\text{cos}}\beta_{0}^{2} } \right)}}$$

When the geometric error of the ball screw sub is not considered, the load magnitude of the ball is converted into the contact stress between the ball and the raceway by Eq. (22), and the fatigue life of the ball screw sub under the action of axial load, coupling load and moment is calculated by Eq. (26).

$$\left\{ {\begin{array}{*{20}c} {M_{Z} = \frac{{\sigma_{0}^{m} N_{0} D_{0} D}}{{60 \times 1396.1^{m} N_{j} \left( {D_{0}^{2} - D^{2} \cos \beta_{0}^{2} } \right)}} = 16423\left( {\text{h}} \right)} \\ {M_{O} = \frac{{\sigma_{0}^{m} N_{0} D_{0} D}}{{60 \times 1563.7^{m} N_{j} \left( {D_{0}^{2} - D^{2} \cos \beta_{0}^{2} } \right)}} = 8340\left( {\text{h}} \right)} \\ {M_{J} = \frac{{\sigma_{0}^{m} N_{0} D_{0} D}}{{60 \times 1608.4^{m} N_{j} \left( {D_{0}^{2} - D^{2} \cos \beta_{0}^{2} } \right)}} = 7024\left( {\text{h}} \right)} \\ \end{array} } \right.$$

**Figure 12:**
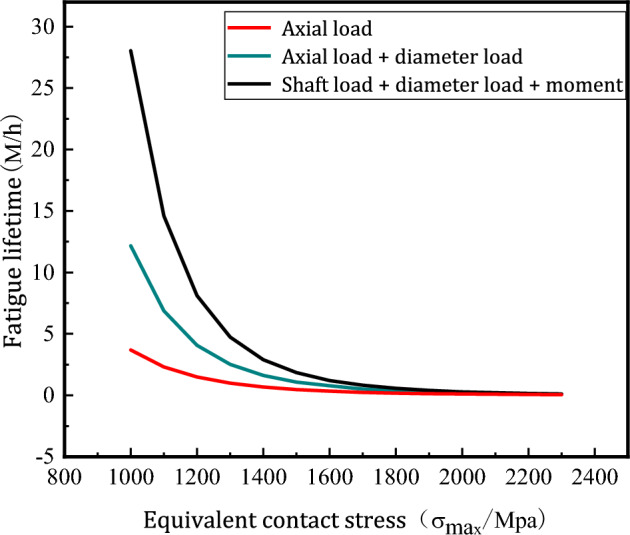
Effect of different loading forms on fatigue life.

As can be seen from Fig. 12, the fatigue life of the ball screw decreases gradually when axial load, coupling load and torque are applied. When the equivalent contact stress is small, the fatigue life of the three different loading forms corresponding to the ball screw sub differs greatly, and the fatigue life decreases sharply when the equivalent contact stress initially increases. According to the analysis of the load distribution of the balls in the raceway, it can be obtained that the coupling load and the moment have different degrees of negative influence on the fatigue life of the ball screw sub, among which the moment has the greatest influence on the fatigue life of the ball screw sub, followed by the influence of radial load. Therefore, in order to prolong the fatigue life of the ball screw sub, it is necessary to improve the accuracy of the ball screw sub during the assembly process, prevent the generation of the tilt angle between the axes of the screw, and reduce the influence of the radial load and the additional rotation time.

### Effect of error on load distribution and ball fatigue life

**Figure 13:**
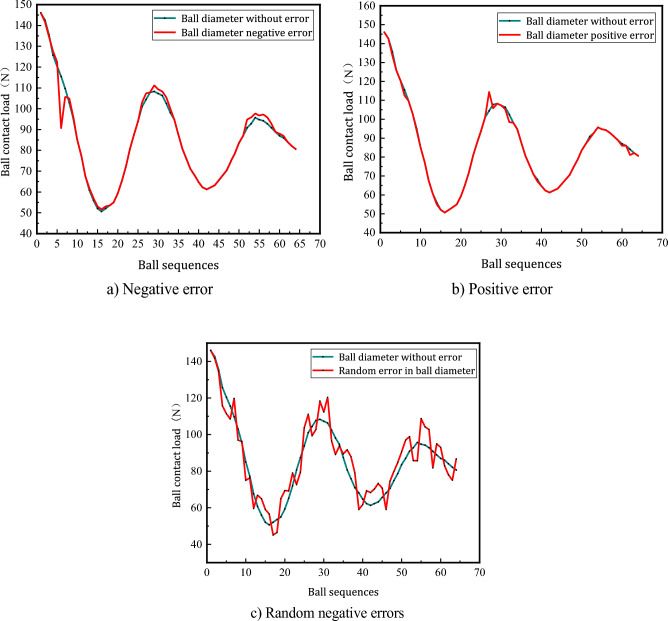
Effect of different ball size errors on load distribution.

Figure 13 shows the ball load distribution of the ball under the influence of different geometric errors.

Figure 13a shows that when the 5th ball has a negative error (− 1.5 um), the contact load increases because the raceway first contacts the larger ball and the contact load increases. Therefore, the load on the ball with negative errors is smaller than when there is no error, and the ball in the return device is not subjected to load.

Figure 13b shows the 27th ball with a positive error (+ 1.5 um), which is generated because the raceway will first contact the larger ball. Therefore, the load on the ball with a positive error is greater than the load on the ball with no error. Figure 13c shows the distribution of the ball load under the influence of randomly generated error within ($\pm \;2\;\;{\text{um}}$), and the load distribution curve shows irregular fluctuations because the error is random.

According to the research results, it is found that the life of the ball screw pair is affected by the size error of the ball. Specifically, when the size error of the fifth ball is (− 1.5 um), the life of the ball screw pair will be shortened by 1030 h. Further analysis shows that this life shortening is caused by the decrease of the contact load of the fifth ball and the increase of the contact load of other balls. The size error leads to incomplete contact between the fifth ball and the ball screw pair, so that the load it bears is reduced, while other balls need to bear greater load.

From the Fig. 13b, when the ball 27 has a positive error (+ 1.5 um), according to the relationship between the contact load and equivalent contact stress (3.22), the corresponding equivalent contact stress can be found as $\sigma_{1.5} = 1493.1\;\;{\text{MPa}}$, $\sigma_{N} = 1462.7\;\;{\text{MPa}}$, and the fatigue life of the ball screw sub can be obtained as:

$$\left\{ {\begin{array}{*{20}c} {M_{ + 1.5} = \frac{{\sigma_{0}^{m} N_{0} D_{0} D}}{{60 \times 1493.1^{m} N_{j} \left( {D_{0}^{2} - D^{2} \cos \beta_{0}^{2} } \right)}} = 10975\left( {\text{h}} \right)} \\ {M_{N} = \frac{{\sigma_{0}^{m} N_{0} D_{0} D}}{{60 \times 1462.7^{m} N_{j} \left( {D_{0}^{2} - D^{2} \cos \beta_{0}^{2} } \right)}} = 12453\left( {\text{h}} \right)} \\ \end{array} } \right.$$

According to the calculation of ball screw fatigue life, when the 27th ball has dimensional error (+ 1.5 um), its own contact load increases and the fatigue life of the ball screw sub is shortened by 1478 h.

By comparing the fatigue life of the ball screw sub under different working conditions, we can see that when there is dimensional error, the fatigue life of the ball screw sub will be significantly reduced.

**Figure 14:**
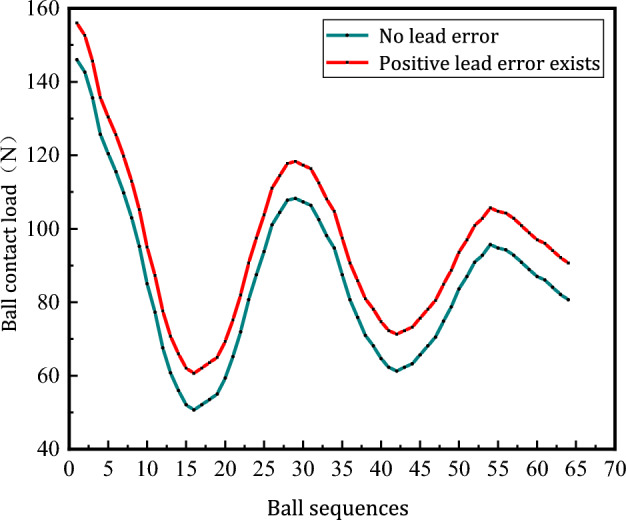
Effect of guide error on ball load distribution.

As shown in Fig. 14, comparing the load distribution of the balls with and without the lead error of the screw, when there is a lead error (+ 1.5 um), the contact load of the balls increases and the maximum equivalent contact stress is $\sigma_{1.5} = 1652.9\;\;{\text{MPa}}$,${ }\sigma_{N} = 1590.5\;\;{\text{MPa}}$. Life is calculated by Eq. (26):

$$\left\{ {\begin{array}{*{20}c} {M_{ + 1.5} = \frac{{\sigma_{0}^{m} N_{0} D_{0} D}}{{60 \times 1652.9^{m} N_{j} \left( {D_{0}^{2} - D^{2} \cos \beta_{0}^{2} } \right)}} = 5963.1\left( {\text{h}} \right)} \\ {M_{N} = \frac{{\sigma_{0}^{m} N_{0} D_{0} D}}{{60 \times 1590.5^{m} N_{j} \left( {D_{0}^{2} - D^{2} \cos \beta_{0}^{2} } \right)}} = 7511.9\left( {\text{h}} \right)} \\ \end{array} } \right.$$

According to the calculated ball fatigue life, it can be seen that when there is lead error (+ 1.5 um), the contact load of all balls increases overall and the life of the ball screw sub is shortened by 1548 h.

Comparing the fatigue life of the ball screw sub under different working conditions, when considering the effect of ball size error on fatigue life, the fatigue life is obviously reduced due to the difference in ball size and the different contact between ball and raceway. Comparing the uneven distribution of ball load caused by lead error and ball size error, the reduction of ball screw sub fatigue life caused by lead error is greater than the reduction of ball screw sub fatigue life caused by ball size error.

The fatigue life of the ball screw sub depends on the ball with the largest contact load of all ball load distributions. For a given ball size accuracy, the fatigue life of a ball screw decreases with increasing external load. By comparing the fatigue life when the ball load is uniformly distributed and when the load is unevenly distributed, it can be seen that the fatigue life is significantly shortened when considering the uneven ball load distribution. Therefore, improving the machining accuracy as much as possible and reducing the influence of geometric error during the machining process can effectively prolong the service life of the ball screw.

The load of the ball screw during the working process will lead to the contact stress between the ball and the raceway. Different load distribution will lead to uneven distribution of contact stress between the ball and the raceway, thus affecting the fatigue life of the ball screw. The ball screw will produce certain deformation in the working process. Different load distribution will lead to different deformation of ball screw, which will affect its working accuracy and stability. If the deformation of the ball screw is too large, the contact state between the ball and the raceway will change, which will affect the fatigue life of the ball screw. Rolling friction will occur in the working process of ball screw, and different load distribution will lead to different rolling friction. Rolling friction will produce friction heat, which will cause the temperature rise of the ball screw, thus affecting its fatigue life.

## Conclusion

Study the actual load applied to each ball in the raceway, analyze the influence of coupling load, rotation time and error on ball load distribution and ball fatigue life, and draw the following conclusions:

1. From the process of single load, coupling load and moment action, the contact load of the ball gradually increases; when the axial load is constant, the load on the ball will show periodic changes with the change of radial load; when the coupling load is certain, the contact load of the ball is not uniform and the amplitude increases during the moment action. Simulation comparison verifies the rationality of the load distribution model.
2. The effect of rotation time on fatigue is greater than the effect of coupling load. In order to improve the fatigue life of the ball screw sub, the assembly accuracy should be improved and a reasonable installation method should be selected to avoid the influence of additional rotating torque.
3. When considering the influence of geometric error on ball contact load, the influence of guide error on ball contact load is greater than the influence of ball size error on ball contact load, and when considering the influence of error, the fatigue life of ball screw sub is shortened. In order to prolong the service life of the ball screw sub, the influence of geometric error should be reduced and the machining accuracy should be improved.

## Data Availability

Since the collection, collation and dissemination of data require a lot of time and resources, and the publication of these data may not produce sufficient benefits to make up for its costs, the data sets generated and/or analyzed in the current study are not publicly available, but can be obtained from the reasonable requirements of the corresponding authors.
